# Migraine-Related Burden, Treatment Interventions, and Healthcare Experiences by Race and Ethnicity: Results of the Migraine Report Card Survey

**DOI:** 10.1007/s40615-025-02577-5

**Published:** 2025-08-06

**Authors:** Amaal J. Starling, Dawn C. Buse, Roger Cady, Kevin Lenaburg, Steven Kymes

**Affiliations:** 1 Division of Concussion, Division of Headache, Department of Neurology, Mayo Clinic Arizona, 13400 East Shea Boulevard, Scottsdale, AZ 85259 USA; 2 Albert Einstein College of Medicine, Bronx, NY USA; 3 RK Consults, Ozark, MO USA; 4 Missouri State University, Springfield, MO USA; 5 Nuvie Bio, San Diego, CA USA; 6 Clusterbusters, Inc, Lombard, IL USA; 7 Lundbeck LLC, Deerfield, IL USA

**Keywords:** Migraine, Headache, Medication overuse, Patient perspective, Harris Poll, US-based survey

## Abstract

Migraine studies consistently have low enrollment of non-White and/or Hispanic participants. This exploratory analysis assesses burden, treatment interventions, and care in people with high-frequency migraine and medication overuse (HFM + MO) in three racial/ethnic groups in the United States that responded to the Migraine Report Card online survey. The Migraine Report Card survey was fielded to adults (≥ 18 years). Eligible respondents reported current or previous HFM + MO (≥ 8 days/month with headache and ≥ 10 days/month of acute headache medication use over the last few months) and screened positive for migraine using the ID Migraine™ screener. Survey questions pertained to living with migraine, healthcare-patient communication, and treatment use and access. Subgroups large enough for analysis included non-Hispanic White, non-Hispanic Black, and Hispanic respondents currently experiencing HFM + MO. Acute medication optimization was assessed with the 4-item Migraine Treatment Optimization Questionnaire. Raw data were weighted to the US adult population. A total of 414 respondents currently experiencing HFM + MO were included in this analysis (White, *n* = 293; Black, *n* = 46; Hispanic, *n* = 75). In this population, despite similarities in migraine and insurance status, 54% of Black respondents reported being “very concerned” about their current health compared to 29% of White and 24% of Hispanic respondents. Twenty percent of White respondents reported using a preventive prescription migraine medication compared to 7% of Black and 10% of Hispanic respondents. Despite similar clinical and socioeconomic characteristics, healthcare disparities were observed, most notably among Black and Hispanic respondents experiencing HFM + MO. Further research regarding inequities in migraine care and disease impact is needed.

## Introduction

Migraine is a common neurological disease that affects approximately 1.3 billion people globally [1]. Migraine attacks encompass several phases and can include many symptoms, including headache, nausea/vomiting, sensitivity to light and sound, cognitive impairment, and fatigue [2, 3], all of which can affect activities of daily life [4]. While migraine is most often episodic and sporadic, some people experience a higher frequency of migraine, which is associated with additional disability, comorbidities, and worse quality of life [3]. When properly diagnosed and with proper management strategies, migraine is a treatable condition [5]. There are two main types of treatment: (1) acute migraine treatment, which aims to eliminate or reduce pain and symptoms once an attack has begun, and (2) preventive migraine treatment, which can prevent migraine attacks from happening and reduce severity, duration, and the impact of the attacks that occur [5].

Migraine is under-diagnosed and often under-treated, with many patients facing barriers to care such as a lack of access to a healthcare professional who has the time and expertise to diagnose and treat migraine; this can create lengthy waits for proper diagnosis and management strategies [6, 7]. Moreover, there can be healthcare disparities or differences in the quality of health and/or healthcare across racial, ethnic, and socioeconomic groups which are not due to access-related factors, clinical needs, or preferences [8]. Healthcare disparities have been documented in a wide range of different aspects of healthcare and disease states [9]. This publication focuses on self-reported details from study participants of different racial and ethnic groups with high frequency migraine (HFM; ≥ 8 days per month) and medication overuse (MO; ≥ 10 days/month of acute medication use) from the Migraine Report Card survey, a US-based survey that explored the life and healthcare experiences of adults with HFM + MO [10].

The American Migraine Prevalence and Prevention (AMPP) study found that adults with high-frequency migraine/headaches were 12 times more likely to have severe disability than those with a lower frequency of migraine/headaches [11]. Moreover, a higher frequency of migraine/headaches was associated with adults being less likely to be employed or to have higher work productivity impairment when compared to those with a lower frequency of migraine/headaches [11, 12]. People with a high frequency of migraine/headaches have high treatment needs and often use acute medication to treat their migraine/headache and related symptoms, which can result in medication overuse and medication overuse headache [13, 14]. The International Classification of Headache Disorders, 3rd edition (ICHD-3) defines medication overuse as use of triptans, ergot alkaloids, combination analgesics, or opioids on 10 or more days per month or use of simple analgesics, including non-steroidal anti-inflammatory drugs on 15 or more days per month [3].

Acute migraine medication overuse is associated with disease worsening that is no longer sufficiently relieved by acute medication (including medication overuse headache and/or progression from episodic to chronic migraine). Moreover, high acute medication use has been shown to be associated with increased depression, anxiety, headache frequency, and headache-related disability [14]. An analysis of the US population-based Chronic Migraine Epidemiology and Outcomes Study (CaMEO) found that medication overuse was more prevalent among Black respondents [7]. This could be a reflection of non-optimized acute medication, lack of preventive treatment options, or self-treatment with over-the-counter medications, which may be due to barriers such as insurance limitations, geography, or affordability, among other hypotheses [7].

Other US studies have highlighted that differences in migraine burden can be exacerbated for certain ethnic, racial, and socioeconomic groups [7]. Some of these differences have been attributed to socioeconomic and/or healthcare disparities, especially in Black and Hispanic populations. Cultural differences and non-optimized communication between healthcare professionals and patients of color are an important but often overlooked source of miscommunication that can impact both medical decision-making and clinical outcomes [15–18]. Moreover, age, gender, sexual orientation, education level, income, family structure, foreign- versus native-born status, refugee or immigrant status, and other social determinants of health can have an effect on individuals’ overall well-being [15, 18].

Despite migraine being one of the most prevalent neurological conditions, with similar prevalence across different racial, ethnic, and geographic populations in the US and worldwide, migraine clinical trials consistently have a lower/non-representative enrollment of non-White racial and/or Hispanic ethnic groups [19–22]. This lack of representation can lead to compromised generalizability of clinical research findings and compounded health disparities in already underrepresented populations [23]. The Harris Poll Migraine Report Card survey had racial and/or ethnic representation to mimic that of the United States (US) 2021 census [10]. Unlike previously performed population-based studies, this survey highlighted the experiences of those currently living with HFM + MO and those who previously had HFM + MO with the hope of identifying factors such as medical care differences, behavioral, or lifestyle changes that may have accounted for improvements in HFM + MO. This present exploratory subgroup analysis of the Migraine Report Card survey was conducted to identify any racial/ethnic disparities in the self-reported healthcare experiences of people with HFM + MO.

## Methods

### Study Design

This is an exploratory subgroup analysis of the Migraine Report Card study, a cross-sectional online survey fielded by the Harris Poll and available to a panel from December 9, 2021, to January 10, 2022. The full study design has been previously published [10]. In brief, the survey took ~ 15 min to complete and consisted of closed-ended questions. Respondents provided electronic informed consent prior to screening and were asked to read/agree to the Harris Poll privacy policy before continuing. This non-interventional survey was not intended to provide clinical data for treatment decisions and was not conducted as a clinical trial. Moreover, no personally identifiable information of respondents was shared with The Harris Poll, the sponsor, or manuscript authors; therefore, Institutional Review Board approval was not sought. Survey respondents were compensated for their time/participation with loyalty points toward panel membership.

### Respondents

To participate in this survey, respondents had to be  ≥ 18 years of age (no upper age limit) and live in the US. Eligible survey respondents were those who had a high probability of having migraine based on self-reported ID Migraine™ screener responses [24]. ID Migraine™ is a validated 3-item screener that identifies individuals who likely have migraine if they answer “yes” to at least 2 of the 3 items [24]. The items ask whether headache has limited activities for ≥ 1 day within the past few months, whether nausea is experienced during headache, and whether there is light sensitivity during headache.

Respondents were classified as having either “current HFM + MO” or “previous HFM + MO.” Current HFM + MO was defined as experiencing ≥ 8 days or parts of days/month with headache or migraine within the past few months and ≥ 10 days/month of any acute headache medication use within the past few months. Previous HFM + MO was defined as previously experiencing the thresholds for current HFM + MO when their headache pattern was at its worst and now experiencing ≤ 7 days or parts of days/month with migraine within the past few months and ≤ 9 days/month of any acute headache medication use within the past few months. In this analysis, HFM + MO was not synonymous with high-frequency episodic migraine (defined in other studies as either 10–14 or 8–14 headache days/month), chronic migraine (defined as headache on ≥ 15 days per month, with 8 of them having migraine symptoms, for ≥ 3 months), or medication-overuse headache, although the respondents could likely be classified into one or more of those groups.

Respondents self-identified their race and ethnicity (combined) by selecting one or more options, which were then combined into subgroups: White, not Hispanic (“White”); Black, not Hispanic (“Black”); Hispanic (of any race); Asian; Native American or Alaskan; Native Hawaiian or Pacific Islander; or more than one race. Analyses presented here were limited to non-Hispanic White, non-Hispanic Black, and Hispanic respondents *currently* experiencing HFM + MO due to smaller sample sizes within previous HFM + MO and other racial/ethnic subgroups that preclude formal comparisons.

### Survey Assessments

The Migraine Report Card survey included screening questions to assess age, geographic location, headache history/characteristics, over-the-counter and/or prescription medication use for migraine/headache, and likelihood of migraine by using the ID Migraine™ screener [24]. Screening also captured demographics (gender, race, and ethnicity), employment status, ratings of overall health, and comorbidities. The survey assessed the following self-reported experiences: diagnosis, healthcare provider (HCP) relationships and experiences, symptom frequency and impact, disease burden and concerns, disease management goals, treatment optimization, perceived stigma, and experience of current/past acute and preventive prescription treatments. Some of these data are published in separate manuscripts [10, 25]. In this survey, a respondent’s HCP referred to the main HCP who treated them for migraine/headaches.

Respondents were asked whether they used over-the-counter medications (acetaminophen, nonsteroidal anti-inflammatories), acute (barbiturates, dihydroergotamine, diclofenac, ditan, ergotamines, gepants, opioids, or triptans), or preventive prescription medications (anti-epileptics, calcitonin gene-related peptide agonists, onabotulinumtoxinA), over-the-counter supplements (e.g., magnesium, B2, ginger, Coenzyme Q10 [CoQ10], green tea, ginseng), or other types of treatments (cognitive behavioral therapy, massage therapy, herbs, marijuana/cannabis [THC]; or cannabidiol [CBD] etc.) to manage their migraine/headaches. Respondents who affirmed using acute treatments (e.g., currently did/used/took something to treat a migraine attack at the time of a migraine/headache attack) were prompted to answer the 4-item Migraine Treatment Optimization Questionnaire (mTOQ-4); a validated subset of the mTOQ-6 used to assess treatment efficacy optimization. The questions on the mTOQ-4 include: (1) “After taking your migraine medication, are you pain free within 2 h for most attacks?”; (2) “Does one dose of your migraine medication usually relieve your headache and keep it away for at least 24 h?”; (3) “Are you comfortable enough with your migraine medication to be able to plan your daily activities?”; and (4) “After taking your migraine medication, do you feel in control of your migraines enough so that you feel there will be no disruption to your daily activities?” Answers and scores to each question were: never (0), rarely (0), less than half the time (1), and half the time or more (2). A score of 0 = very poor treatment efficacy, 1–5 = poor treatment efficacy, 6–7 = moderate treatment efficacy, and 8 = maximum treatment efficacy [26].

### Data Collection and Analysis

A full report of data collection and analysis can be found in the Migraine Report Card primary publication [10]. In brief, no formal power calculations were conducted. The sample size was determined based on the feasibility of the online panel and the appropriate balance to be able to compare between different groups. The overall quota was set at *n* = 400 (current HFM + MO) and *n* = 100 (previous HFM + MO). To ensure this survey was representative of the US population, the sample group used for weighting was the total sample of US age ≥ 18-years respondents. All raw data were weighted via the Random Iterative Method (RIM) where necessary to align them with their actual proportions in the US population from benchmarks from the March 2021 US Census Bureau’s Current Population Survey Annual Socioeconomic Supplement [27]. They were weighted by age (18 +), education, gender, race, Hispanic ethnicity, US Census region, household income, household size, and marital status. In this analysis, unweighted sample sizes are presented; however, all percentages displayed were calculated using weighted data. Because of this, there are no numerators or denominators presented.

Analyses reported in this manuscript were limited to patients currently experiencing HFM + MO due to smaller sample sizes in subgroups within the previous HFM + MO group (White, *n* = 91; Black, *n* = 4; Hispanic, *n* = 5), which precluded formal comparison. Descriptive statistics included mean and standard deviation for continuous variables and percentage rates for categorical variables. Statistically significant differences between the current and previous groups were determined by a standard *t* test of column proportions and means at the 90% (*P* < 0.1) and 95% (*P* < 0.05) confidence levels; however, comparisons were not controlled for multiplicity. The sampling precision was measured by using a Bayesian credible interval; for this survey, the sample data are accurate to within ± 5.3 percentage points using a 95% confidence level. Data were analyzed using IBM® Quantum, version 5.8 (IBM Corporation, Armonk, NY, USA).

This survey required respondents to answer each question (and any additional sub-questions) before moving to the next question; therefore, there were no missing data. Questions of a sensitive nature allowed respondents to select “prefer not to answer”; however, this percentage was relatively low across different questions.

## Results

### Survey Population and Demographics

A total of 414 US adults were categorized into the current HFM + MO group. The racial and ethnic breakdown of respondents was 293 (70.7%) White respondents, 46 (11.1%) Black respondents, and 75 (18.1%) Hispanic respondents. Gender representation was generally balanced between all groups and enriched for males, with about 10% more females compared to males in each group (Table 1). Black respondents and Hispanic respondents were generally younger than White respondents (38.5 years and 36.7 years vs. 43.8 years, respectively). White respondents had slightly more headache days per month when compared to Black or Hispanic respondents (mean 15.4 days vs. 14.9 days and 14.3 days, respectively). Sixty-one percent of Hispanic respondents reported having received a diagnosis of chronic migraine compared to 42% of Black respondents and 49% of White respondents. The percentage of insured respondents was ≥ 91% across all three racial/ethnic groups; however, the types of insurance varied per group, with Black and Hispanic respondents reporting a larger percentage of employer-provided commercial insurance (respondents’ employer or family members’ employer; 54% and 66%, respectively) compared to White respondents (42%). Twenty-one percent of White and 19% of Hispanic respondents reported being covered by Medicare compared to 5% of Black respondents. Seventy-two percent of Black respondents reported being employed full time compared to 51% of White respondents and 59% of Hispanic respondents; 13% of White respondents reported being not employed/unable to work due to a disability or illness and 10% reported being retired compared to 3% and 1% of Black respondents, respectively, and 4% and 4% of Hispanic respondents, respectively.

**Table 1:** Demographics and clinical characteristics

	White (not Hispanic [W]) *n*=293	Black (not Hispanic [B]) *n*=46	Hispanic [H] *n*=75
Gender, %			
Male	45%	38%	45%
Female	54%	59%	55%
Non-binary or gender non-conforming	1%	2%	0%
Prefer not to answer	0%	2%^w^	0%
Age, %			
≤ 50 years	69%	89%^W^	92%^W^
> 50 years	31%^B,H^	11%	8%
Mean (SD)	43.8 (13.0)^B,H^	38.5 (11.5)	36.7 (11.3)
Mean age at first migraine (SD)	22.5 (11.6)	23.1 (11.1)	20.3 (9.5)
Mean age at first diagnosis of migraine/headache (SD)	25.1 (12.2)^H^	26.2 (11.0)	21.0 (8.9)
Highest level of education, %			
Less than high school	11%	5%	8%
High school to less than 4-year college degree	52%	62%	60%
4-year college degree or more	37%	33%	31%
Health insurance type, %			
Employer provided	31%	49%^w^	43%
Medicare	21%^B^	5%	19%^b^
Medicaid	23%^H^	29%^H^	11%
Provided by family member’s employer	11%	5%	23%^W,B^
Individual insurance policy bought by self/family member	11%	17%	10%
From an exchange (i.e., marketplace)	13%	12%	8%
Veterans’ benefits	2%	0%	1%
Health insurance provided to students (via college/university)	2%	0%	6%^w^
Other	1%	0%	0%
None	7%	9%	6%
Employment Status, %			
Employed full time	51%	72%^W^	59%
Employed part time	6%	7%	4%
Self-employed full time	3%	1%	3%
Self-employed part time	2%	2%	1%
Not employed, but looking for work	7%	12%	11%
Not employed and not looking for work	0%	0%	0%
Not employed, unable to work due to a disability or illness	13%^b,h^	3%	4%
Retired	10%	1%	4%
Student	1%	0%	8%^W^
Stay-at-home spouse or partner	7%	2%	6%
Total household income, %			
Less than $15,000	5%	9%	10%
$15,000 to $24,999	9%	10%	6%
$25,000 to $34,999	10%	5%	7%
$35,000 to $49,999	12%	4%	5%
$50,000 to $74,999	16%	22%	25%
$75,000 to $99,999	10%	28%^W,H^	8%
At least $100,000	35%	22%	39%
Prefer not to answer	2%	0%	0%
Size of household, %			
1 member	8%	22%^W^	10%
2 members	25%^h^	18%	13%
3 members	19%	28%	28%
4 members	28%^b^	12%	24%
5+ members	21%	20%	25%
Number of headache days in last few months			
1–7 days/month, %	0%	0%	0%
8–10 days/month, %	36%	29%	35%
11–14 days/month, %	12%	18%	21%^w^
15 days/month, %	16%	19%	15%
16–19 days/month, %	7%	6%	9%
20 days/month, %	12%	18%	9%
≥ 21 days/month, %	16%	10%	11%
Mean (SD)	15.4 (6.2)	14.9 (4.6)	14.3 (4.9)
Number of treatments ever used to treat migraine/headache			
1–3 treatments, %	38%	55%^w^	37%
4–6 treatments, %	34%	29%	45%
7–10 treatments, %	18%	16%	11%
≥ 11 treatments, %	10%^b^	0%	8%
Mean (SD)	5.3 (3.7)^b^	4.1 (2.3)	4.7 (2.8)
Acute medication use in last few months^†^			
≤ 9 days/month, %	0%	0%	0%
10 days/month, %	20%	13%	24%
11–14 days/month, %	11%	18%	27%^W^
15 days/month, %	17%	28%^H^	9%
16–19 days/month, %	8%	4%	12%
20 days/month, %	19%	10%	20%
≥ 21 days/month, %	25%^H^	26%^H^	8%
Mean (SD)	17.9 (6.5)^H^	17.2 (5.9)	15.4 (5.2)

Percentages may not add up to 100% due to rounding. ^**†**^Acute medication use is any over-the-counter or prescription medication to treat headaches. ^w/b/h^Lowercase letters indicate significantly higher than the corresponding group (labeled in the header as W, B, or H) at the 90% confidence level (*p* < 0.1). ^W/B/H^Uppercase/underlined letters indicate significantly higher than the corresponding group (labeled in the header as W, B, or H) at the 95% confidence level *(p* < 0.05). Abbreviation: *SD*, standard deviation

### Overall Health, Concern, and Headache Characteristics

**Fig. 1:**
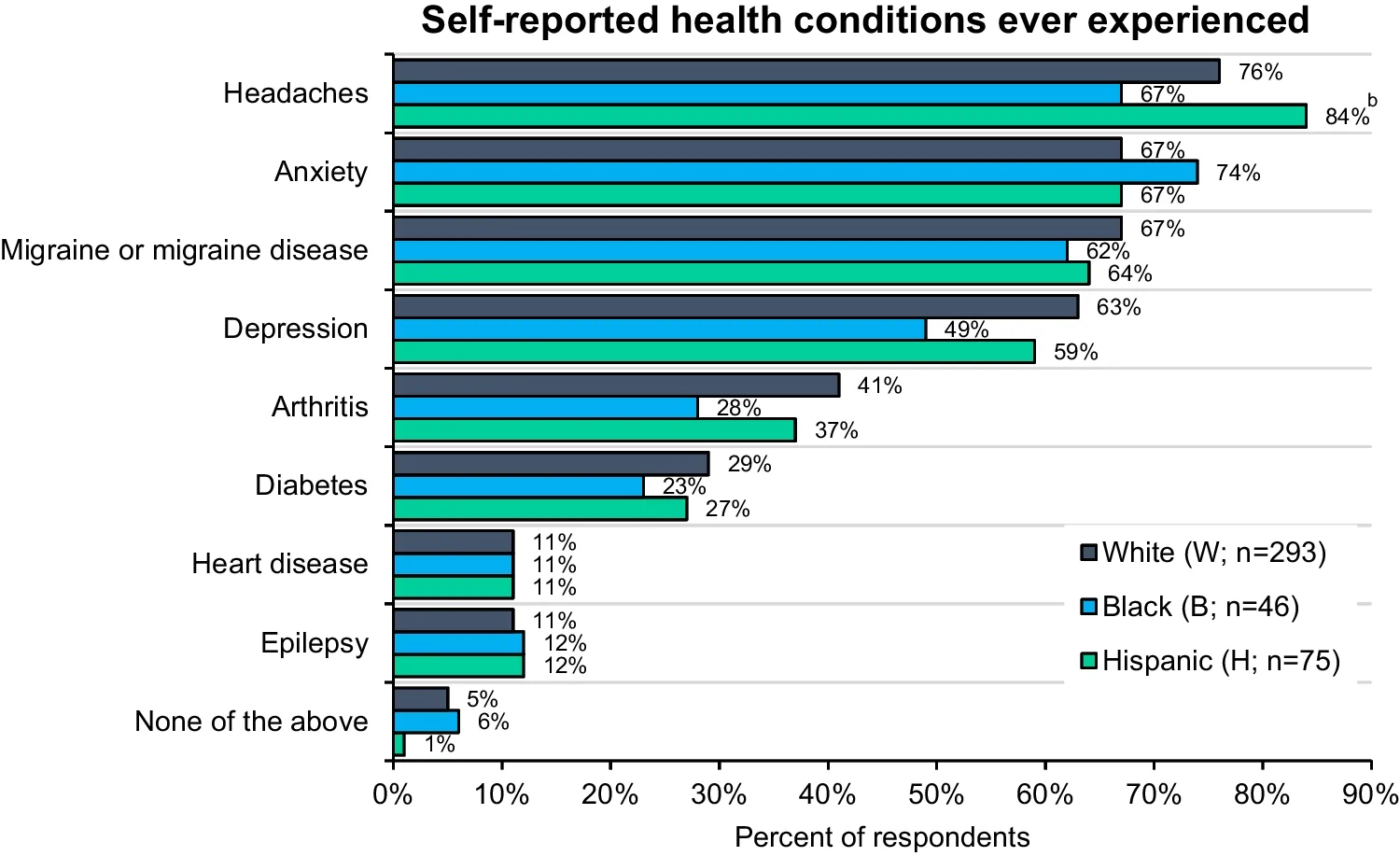
Self-reported comorbidities/health conditions ever experienced. All respondents were given a pre-populated list and asked, “Have you ever experienced any of the following? Please select all that apply.” ^w/b/h^Lowercase letters indicate significantly higher than the corresponding group (labeled in the header as W, B, or H) at the 90% confidence level (*P* < 0.1). ^W/^^B/H^Uppercase/underlined letters indicate significantly higher than the corresponding group (labeled in the header as W, B, or H) at the 95% confidence level (*P* < 0.05)

**Fig. 2:**
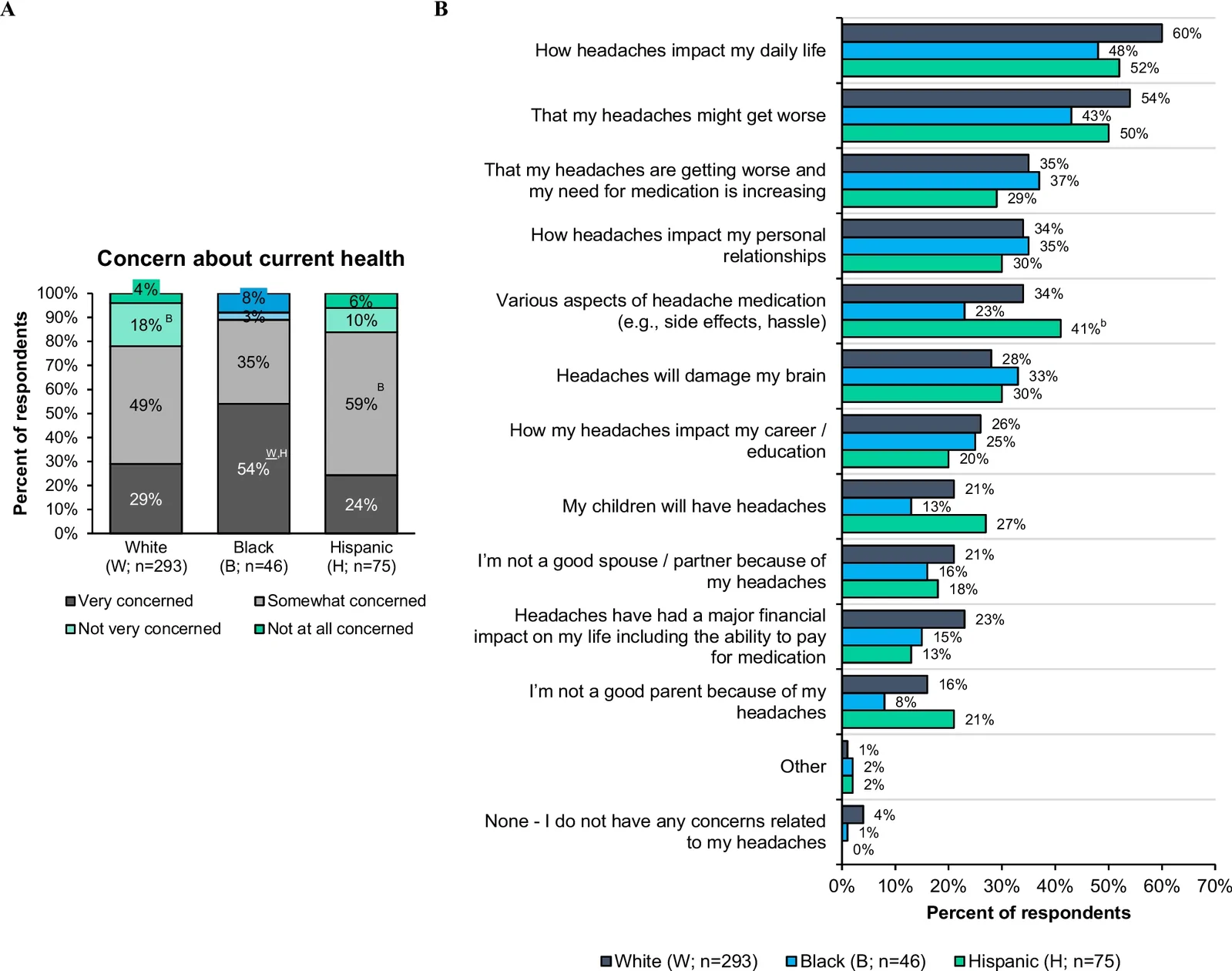
Concern about current overall health (**A**) and current headache concerns experienced (**B**). **A** All respondents were asked: “How concerned are you about your current overall health?” **B** All respondents were asked: “Which of the following are you currently concerned about related to your headaches? Please select all that apply.” ^w/b/h^Lowercase letters indicate significantly higher than the other group at the 90% confidence level (*P* < 0.1) for White, Black, or Hispanic respondents, respectively

Respondents reported having comorbidities and, despite all respondents likely having migraine per the ID Migraine™ screener, only 62–67% of each group reported ever experiencing migraine or migraine disease (Fig. 1). A total of 65% of females and 69% of males reported ever experiencing migraine or migraine disease. When asked to describe current overall health, there were no significant differences by race/ethnicity; however, when asked how *concerned* respondents were about their overall health, 54% of Black respondents reported that they were “very concerned” compared to 29% of White and 24% of Hispanic respondents (Fig. 2A). Respondents expressed current headache concerns, including how headaches impact their daily life, that headaches might get worse, and that current headaches are getting worse and their need for medication is increasing (Fig. 2B). Regarding respondents’ most bothersome symptom other than headache/head pain, 37% of Black respondents reported throbbing/pulsating pain (compared to 11% of Hispanic and 17% of White respondents) and 7% reported allodynia (compared to 0% for both White and Hispanic respondents) as their most bothersome symptom.

### Migraine/Headache Treatment

**Fig. 3:**
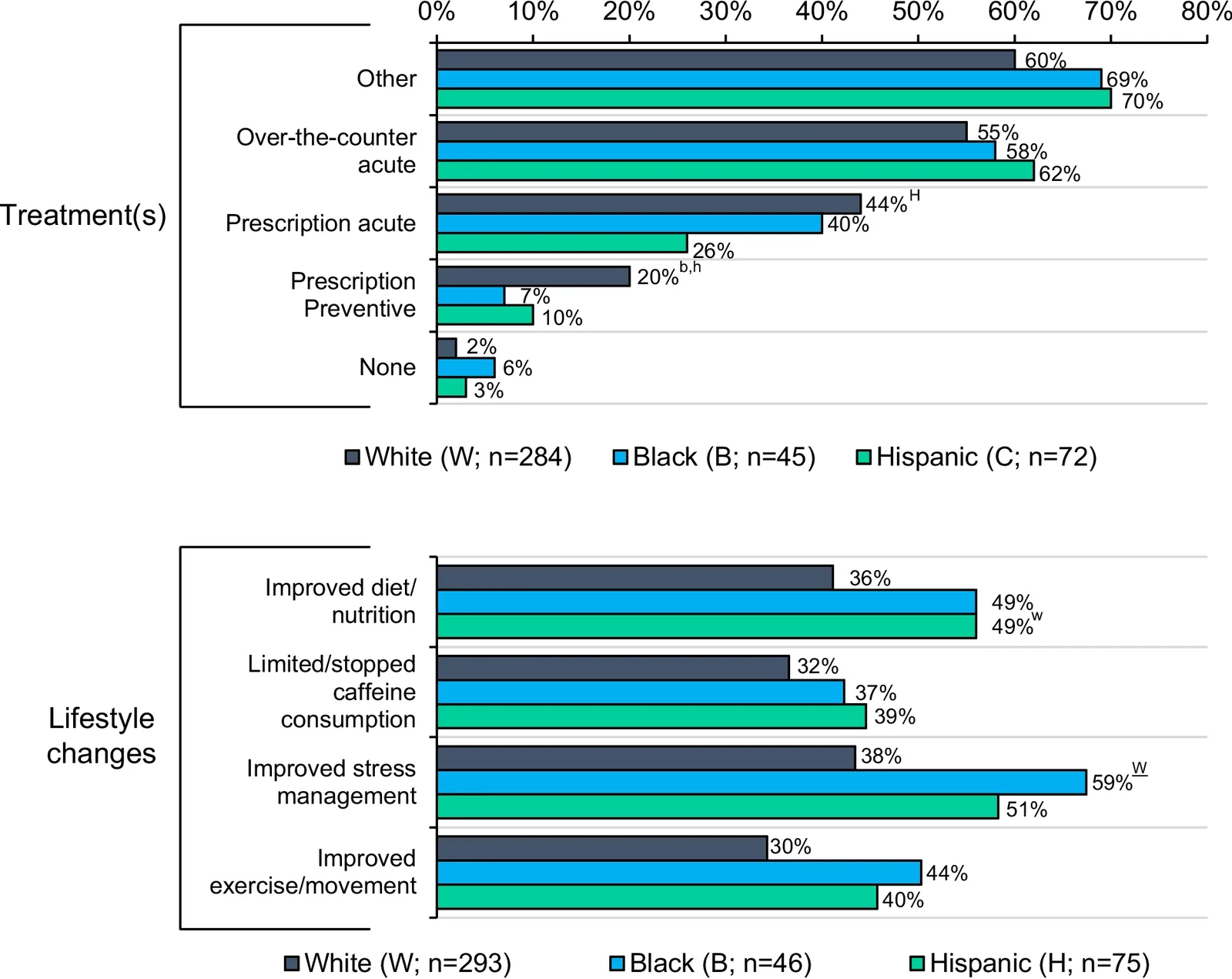
Interventions ever used to manage headaches/migraine. Respondents who had ever used, taken, or done something to treat headache were asked: “Do you currently (or in the past few months) use, take, or do any of the following to treat your headaches? Please select all that apply.” ^w/b/h^Lowercase letters indicate a higher proportion than the corresponding group (legend labels: W, B, H) at *P* < 0.1. ^W/B/H^Uppercase/underlined letters indicate a higher proportion than the corresponding group (axis labels: W, B, H) at *P* < 0.05. The “Other” category included: vitamins, herbs, or other over-the-counter supplements (e.g., magnesium, B2, ginger, Coenzyme Q10, green tea, ginseng); marijuana / cannabis (THC); cannabidiol (CBD); massage therapy; bio-behavioral therapies (cognitive behavioral therapy, biofeedback, relaxation therapies, mindfulness-based therapies); oxygen; chiropractic, physical therapy, or occupational therapy; nerve blocks; oral or dental devices (to correct grinding, clenching, Temporomandibular disorders, apnea, etc.); hormonal birth control or hormone replacement therapy; neuromodulators or neurostimulators (e.g., Cefaly, sTMS mini, gammaCore); other complementary, integrative, or alternative therapy (e.g., acupuncture/dry needling); and other. “Over-the-counter” included: acetaminophen (e.g., Tylenol, Excedrin) and nonsteroidal anti-inflammatory drugs (NSAIDs) (e.g., aspirin, ibuprofen, naproxen sodium, diclofenac). “Acute” medication included: opioids (e.g., oxycodone, Vicodin); triptans (e.g., Imitrex, Maxalt, Zomig, Axert, Relpax, Frova, Amerge); barbiturates (e.g., Amytal, Butisol, Nembutal, Fiornal, Fiorecet); gepants (e.g., Ubrelvy, Nurtec ODT); diclofenac (e.g., Cambia, Flector, Solaraze); dihydroergotamine (e.g., D.H.E. 45, Migranal); ditan (e.g., Reyvow); ergotamines (e.g., Cafergot, Ergomar). “Preventive” medication included: antiepileptic drugs (AEDs) (e.g., Topamax, Zonegram, Keppra); Botox; calcitonin gene-related peptide antagonists (e.g., Aimovig, Ajovy, Emgality, Vyepti)

**Fig. 4:**
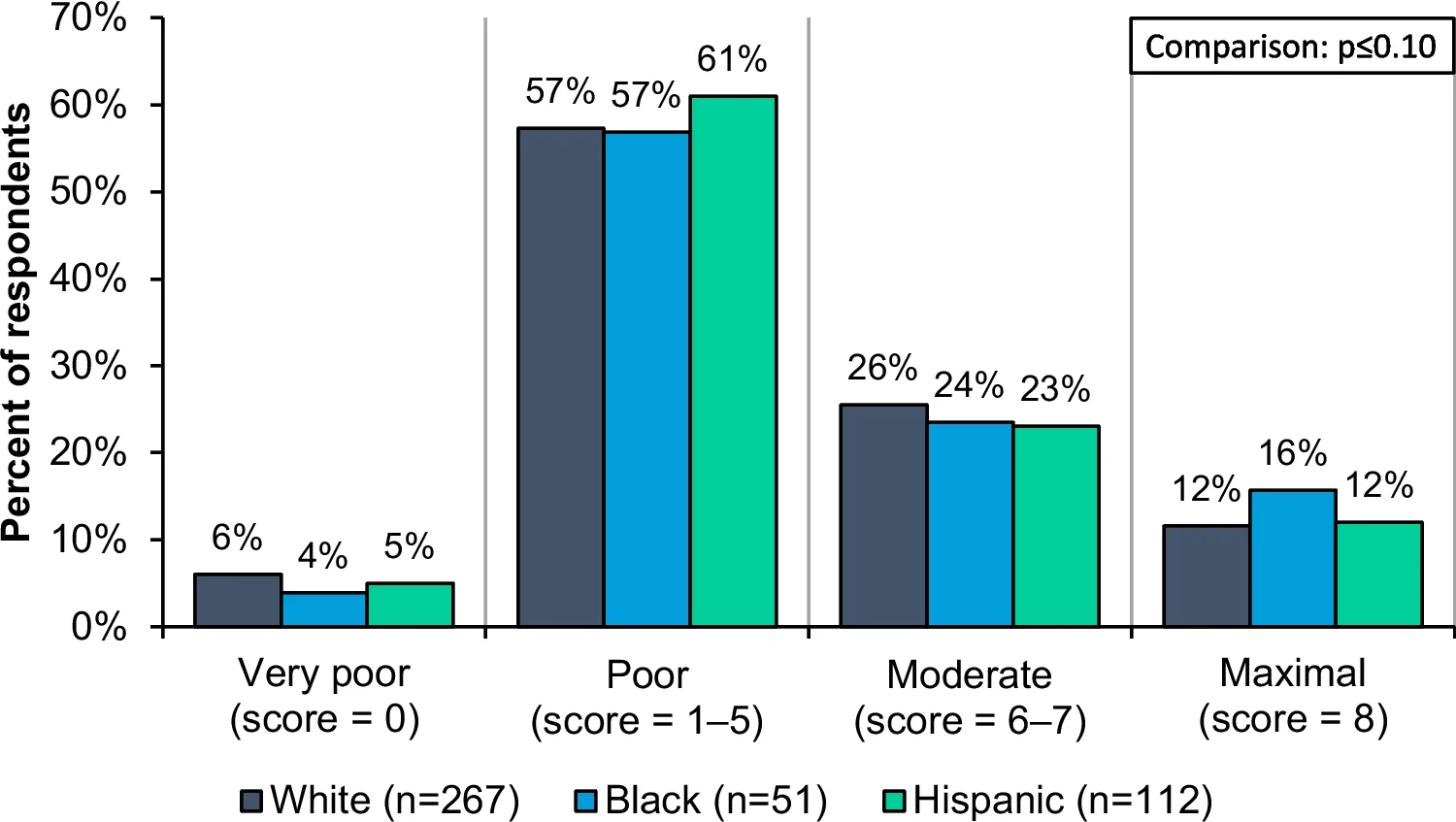
Respondent’s acute migraine treatment optimization assessed by the mTOQ-4. Respondents who had currently (or in the past few months) used, taken, or done something to treat headache were asked: “Please answer the following questions about the medication(s) that you currently use to treat headaches.” Abbreviation: mTOQ-4, 4-item Migraine Treatment Optimization Questionnaire

Ten percent of White respondents reported having tried ≥ 11 acute and/or preventive treatments in their lifetime for their migraine/headaches compared to 0% of Black and 8% of Hispanic respondents. Fifty-five percent of Black respondents reported having only tried 1–3 treatments compared to 38% of White and 37% of Hispanic respondents (Table 1). Over-the-counter medication usage across all groups was similar (55–62%); however, differences were observed with prescription migraine/headache medication usage (acute and preventive medications) where 44% of White respondents compared to 40% of Black and 25% of Hispanic respondents were currently using a prescription acute migraine medication. Similarly, 20% of White respondents compared to 7% of Black and 10% of Hispanic respondents were currently using a preventive prescription migraine medication. Conversely, 69% of Black respondents and 70% of Hispanic respondents used other treatments, including but not limited to items such as vitamins, herbs, or other over-the-counter supplements compared to 60% for White respondents (Fig. 3). Moreover, 49% of Hispanic respondents reported adopting healthier eating/drinking habits to improve headaches compared to 36% of White respondents, and 59% of Black respondents reported making managing stress a priority compared to 38% of White respondents (Fig. 3). Overall, respondents generally had similar acute treatment optimization as assessed by the mTOQ-4 questionnaire, with 57–61% of respondents from each subgroup having “poor” acute treatment optimization (Fig. 4).

### Healthcare Provider Communication

**Fig. 5:**
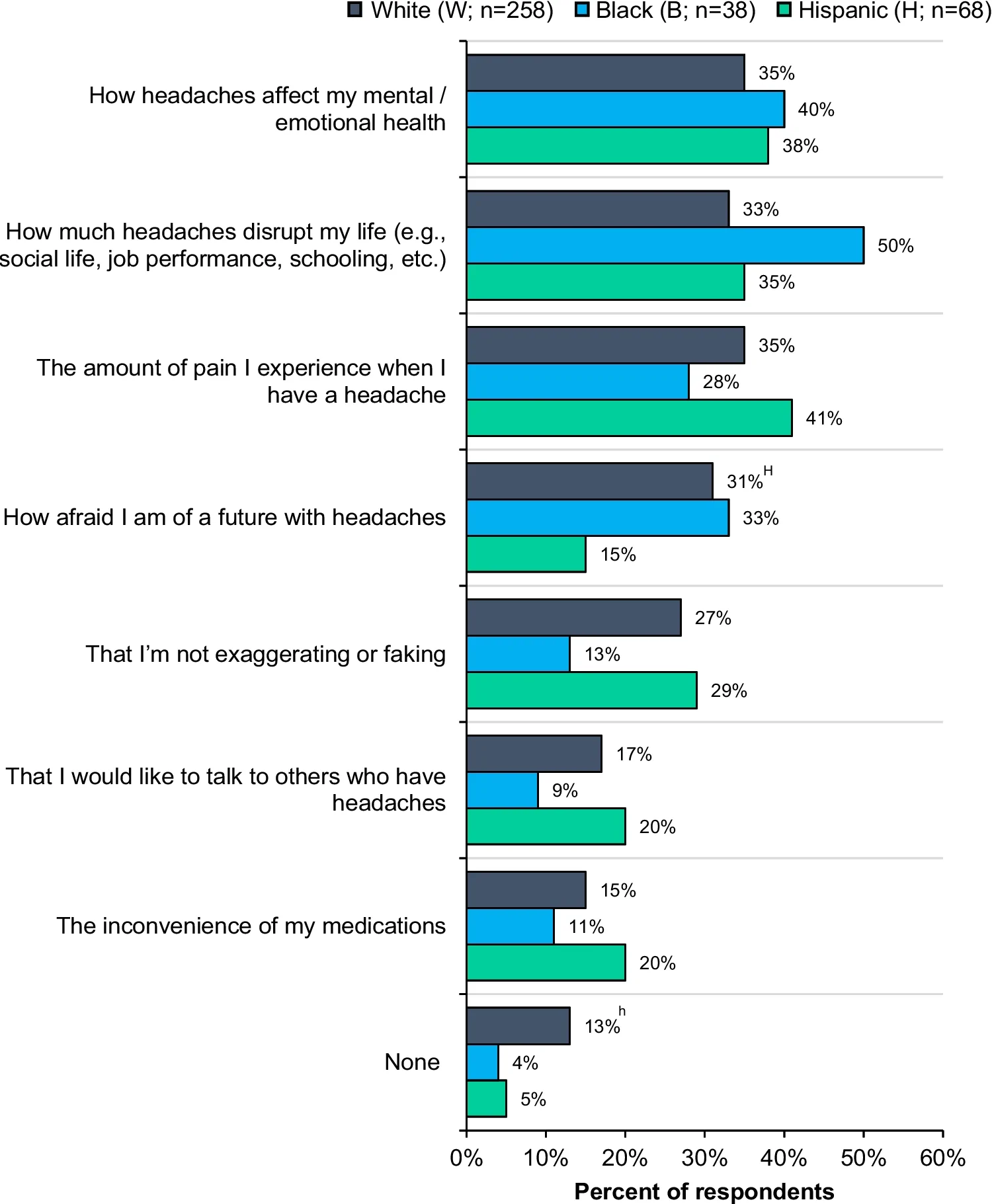
Experiences living with headaches that respondents wished the main healthcare professional who manages their headaches better understood. Respondents currently seeing an HCP to manage their headaches were asked: “Which of the following, if any, do you wish your healthcare provider who currently manages your headaches, better understood about your experiences living with headaches? Please select all that apply.” ^w/b/h^Lowercase letters indicate significantly higher than the other group at the 90% confidence level (*P* < 0.1) for White, Black, or Hispanic respondents, respectively. Uppercase/underlined letters indicate significantly higher than the other group at the 95% confidence level (*P* < 0.05) for White, Black, or Hispanic respondents, respectively

Forty-seven percent of White respondents felt that they made shared treatment decisions together with their HCP compared to 31% of Hispanic and 36% of Black respondents. Similarly, 29% of Hispanic and 41% of Black respondents reported seeking more resources and information about their condition at HCP visits (e.g., assistance programs, patient organizations, support groups) compared to 16% of White respondents. Seventy-two percent of Hispanic respondents and 80% of Black respondents strongly/somewhat agreed that they wished their HCP talked more about their headache management goals compared to 59% of White respondents (Fig. 5).

As a top goal in managing headaches, 14% of Black respondents wished to reduce the level of worry or anxiety they experience about the next headache compared to 3% of White and 2% of Hispanic respondents. Moreover, 12% of Black respondents wished to reduce their need for urgent care/emergency department visits compared to 1% of White respondents.

## Discussion

The Harris Poll Migraine Report Card survey was a US-based survey of adults with HFM + MO. Due to a nationally representative percentage of non-White respondents, this exploratory subgroup analysis allowed self-reported experiences of people with HFM + MO from different racial and ethnic backgrounds to be explored. In the current HFM + MO subpopulation from the Migraine Report Card survey, 57% identified as White, 11% identified as Black, and 24% identified as Hispanic. In previous population-based studies of those with migraine, 79–90% of respondents were White, 7–10% were Black, and 10–13% were Hispanic [11, 28–30]. Overall, the data from this survey showed that respondents had similarities in their migraine history, their comorbidities, and socioeconomics, including health insurance status and type, education, and income level. Despite this, differences were observed in how respondents from these subgroups experience life with migraine/headache, what treatments are used, and their perceptions of their interactions, communication, and relationships with their healthcare providers.

Some similarities observed between respondents in this exploratory data included socioeconomic characteristics such as health insurance, where over 91% of each group was insured (national average as of 2022 was 92% of Americans insured) [31]; education level, where more than 30% of each group had a 4-year college degree (national average as of 2022 was 37.7% of adults had a 4-year degree) [32], and household income, where at least 45% of each group reported earning $75,000 or more per year (median household income was $74,580 in 2022 [33]). Similarly, employment levels were high with over 60% of White and Hispanic respondents and over 80% of Black respondents being employed at least part time. Moreover, in this respondent population, comorbidities were similar across racial and ethnic subgroups. Most notably, anxiety and depression were common comorbidities affecting approximately 50% or more of each group. This finding is similar to findings from other US population-based studies of adults with high-frequency migraine [11, 28]. Similarly, respondents from each group expressed concerns about their migraine/headaches, including how headaches impact their daily life, that headaches might get worse, that current headaches are getting worse, and their need for medication is increasing. Despite respondents having similar access to care based on health insurance status and all respondents likely having migraine per the ID Migraine™ screener, 33–38% of each group reported that they never experienced migraine or migraine disease. This shows there is a national lack of public awareness of migraine which could lead to under consultation, under diagnosis, and under treatment as well as a continued need for education about migraine for both HCPs and the general population.

Differences in experiences were also observed between respondents of different racial/ethnic backgrounds despite having similar sociodemographic factors as discussed above. For instance, when asked to describe current overall health, no major difference was observed by race/ethnicity; however, when asked how *concerned* respondents were about their overall health, Black respondents had a higher rate of reporting “very concerned” compared to White and Hispanic respondents. Differences were also observed in respondents’ symptom reporting where more Black respondents identified throbbing/pulsating pain or allodynia when compared to both White and Hispanic respondents as their most bothersome symptom. Interestingly, a previous questionnaire-based study showed that allodynia, a painful condition where even light touch causes significant discomfort, was more prevalent in Black participants [34].

Some members of non-White ethnic groups have a general mistrust of the medical system, which can be tied to generational and current disparities faced outside and inside of the healthcare system [35]. Despite the changes that have occurred in the US over the past 60 years (since the end of legalized segregation), ethnic/racial discrimination remains and can be felt and observed across all aspects of life, including healthcare [36]. Similarly, race-based traumatic stress and experiences may be passed down from generation to generation, which can influence lived experiences in healthcare settings [37]. Race- and ethnicity-based healthcare disparities have been identified in many areas of healthcare, including the perinatal experience, where Black women are 3–4 times more likely to die from a pregnancy-related complication when compared to White women in the US [38]. Moreover, women of color have reported experiencing perinatal discrimination because of their race/ethnicity, gender, age, marital status, and insurance type [39]. Further, a study regarding prostate cancer in Black men revealed that cultural factors, including mistrust of the healthcare system, poor physician–patient communication, and fear of diagnoses, can lead to racial disparities and poorer healthcare outcomes [40].

A recent cross-sectional study from the Survey of California Adults on Serious Illness and End-of-Life 2019 confirmed that racial/ethnic background was significantly associated with the level of medical mistrust. In this study, the odds of reporting medical mistrust were 73% higher and 49% higher among non-Hispanic Black and Hispanic adults when compared with non-Hispanic White adults, respectively [41]. Additional studies have shown that Black Americans are systematically undertreated for pain relative to White Americans, and false beliefs about biological differences between Black and White patients have been observed among medical professionals, which can contribute to racial disparities in pain assessment and treatment [42]. Similar studies showed that Black and Hispanic patients were less likely than White patients to receive analgesia for acute pain [43].

These examples from the literature display the disparities that can be faced by non-White patients in various aspects of medical care. While it was not fully assessed how healthcare disparities affected the individual respondents in this survey, differences in responses related to migraine care were observed for the different ethnic/racial groups represented here. In this survey, it was shown that Black and Hispanic respondents reported using less prescription acute or preventive migraine/headache medication when compared to White respondents, despite equal access to care indicated by insurance status. Notably, in this patient population, all respondents were likely candidates for preventive care, based upon the recommendations set forth by the American Headache Society [44] and the National Headache Federation Guidelines [45], because of the frequency of their migraine/headache days; however, 20% of White respondents and only 7% of Black and 10% of Hispanic respondents were using a preventive prescription medication. Similarly, a higher percentage of White respondents reported using prescription acute treatment(s) and to have tried ≥ 11 treatments (over-the-counter or prescription acute and/or preventive treatments) in their lifetime for their migraine/headaches, whereas a higher percentage of Black respondents reported having only used 1–3 treatments. The CaMEO study showed that Black respondents were more likely to have acute medication overuse when compared to respondents from other racial backgrounds [7]. Similarly, respondents with a Hispanic background were also more likely to have acute medication overuse when compared to non-Hispanic respondents [7]. Previous studies, combined with what was observed in the Migraine Report Card survey, suggest that non-White racial/ethnic populations may be offered less medication from their HCP or chose not to take offered medication, especially preventive prescription medications that could not only prevent migraine but also reduce the reliance on less effective treatments that are often overused. Inequities including non-optimized acute medication, lack of preventive treatment options, or self-treatment with over-the-counter medications could be caused by barriers such as insurance, geography, or affordability [7].

Overall, this exploratory subgroup analysis supports that migraine healthcare disparities exist for Black and Hispanic respondents when compared to White respondents with HFM + MO, particularly regarding migraine treatment options, despite similar health and socioeconomic status and similar access to care based on health insurance status. It is important to ensure that healthcare professionals acquire effective training in systemic racism, healthcare disparities, implicit bias, and cross-cultural communication to ensure high quality, equitable care is received by all patients. Moreover, support groups and patient organizations can play a critical role in improving the lives of people with migraine from diverse racial and ethnic groups. These organizations can provide culturally relevant education, a sense of community, and a safe space to share experiences—which in turn can help reduce isolation, build trust in healthcare, and address disparities in care and treatment. The relationship between various factors that may lead to healthcare disparities across racial and ethnic populations is complex and must continue to be explored [46].

### Limitations

All data were collected from a pool of people who agreed to participate in online survey research. Therefore, respondents had to have internet access, and all panelists had to complete a “confirmed” or “double opt-in” process to be included. This access may be associated with higher socioeconomic status or employment status, whereas many barriers to care have been found to be associated with lower socioeconomic status for all races and ethnicities. In addition, respondents were required to read and understand English as the survey was not offered in Spanish, which may limit generalizability of these findings to all Hispanic Americans with migraine and may further underestimate health disparities for this group of people. This analysis could only explore responses from those respondents currently experiencing HFM + MO due to smaller sample sizes in subgroups within the previous HFM + MO group; therefore, we could not determine if respondents in the previous group experienced similar disparities. Respondents in this study appear to represent the socioeconomic mean to that of the general US population; however, the higher socioeconomic status of people from underrepresented races and ethnicities in this manuscript may *underestimate* the disparity in experiences for Black and Hispanic individuals compared with White individuals with migraine in the US. This respondent population was highly insured, with ≥ 91% having some type of healthcare coverage; thus, we are unable to fully assess differences based on access to care/insurance, and this highly insured population may not be indicative of the overall US migraine population; this as well may underestimate health access and treatment disparities. The respondent population was not subdivided or analyzed by insurance type (e.g., government vs. commercial); therefore, comparisons between participants based upon insurance type were not determined. This survey does not cover access issues or other disparities. Data were self-reported without supporting documentation or medical records for verification. Although the validated ID Migraine™ screener was used to positively screen respondents for migraine, it is not a diagnostic questionnaire, nor does it replace a clinical interview conducted by a healthcare professional using International Classification of Headache Disorders 3rd edition (ICHD-3) criteria. The ID Migraine™ screener only suggests a likely migraine diagnosis. In a previous publication, it was found to have a pooled sensitivity estimate of 0.84 (95% confidence interval 0.75–0.90) and specificity of 0.76 (95% confidence interval 0.69–0.83) [47]. In addition, survey respondents may have had other headache diagnoses as well. This survey was limited in the number of respondents (414 total); however, data were weighted to be representative of the larger population of US adults with migraine. Because of smaller sample sizes (particularly for Black and Hispanic respondent subgroups), statistical analyses were not set for formal power calculations and were not controlled for multiplicity. Due to the larger than usual for migraine studies sample of males in this study, the overall population may not be representative of the overall migraine population. It was not explored in this study whether Black and Hispanic respondents were offered fewer treatment options or independently chose not to try as many treatments as White respondents; further research on this topic is warranted. White respondents on average were 5–7 years older than Black and Hispanic respondents, respectively. This may account for fewer medications being used by Black and Hispanic respondents as well as other differences observed between these subgroups. Race and ethnicity were not collected and analyzed as separate variables, which may lead to some limitations in comparing data to data from other studies. Hispanic respondents as well as Black respondents were analyzed as one group while these are both in fact heterogenous groups of individuals. They were not divided by race, geographic origin, or other grouping. Non–English-speaking respondents were not included.

## Conclusions

Results from this exploratory subgroup analysis of the Migraine Report Card survey explored disparities in migraine treatment and experiences in respondents with HFM + MO grouped by self-reported race and ethnicity and found gaps in care, especially for Black and Hispanic adults. Despite similarities in migraine health and insurance status, a higher percentage of Black respondents reported being “very concerned” about their current health compared to White and Hispanic respondents. Moreover, a higher percentage of White respondents reported using a preventive prescription migraine medication when compared to Black and Hispanic respondents. While this study was exploratory in nature, we hope that this will continue to drive more research into healthcare disparities as more research regarding migraine-related burden and care in diverse racial and ethnic groups is needed, including how to address disparities, such as inequitable care. HCPs should address these concerns for more equitable care by ensuring the initiation of migraine-specific acute or preventive therapies for patients of diverse racial/ethnic backgrounds and effectively optimizing migraine treatment.

## Data Availability

In accordance with EFPIAand PhRMA’s “Principles for Responsible Clinical Trial Data Sharing” guidelines, Lundbeck is committed to responsible sharing of clinical trial data in a manner that is consistent with safeguarding the privacy of patients, respecting the integrity of national regulatory systems, and protecting the intellectual property of the sponsor. The protection of intellectual property ensures continued research and innovation in the pharmaceutical industry. Deidentified data are available to those whose request has been reviewed and approved through an application submitted to https://www.lundbeck.com/global/our-science/clinical-data-sharing.
